# Parvalbumin-positive interneurons of the prefrontal cortex support working memory and cognitive flexibility

**DOI:** 10.1038/srep16778

**Published:** 2015-11-26

**Authors:** Andrew J. Murray, Marta U. Woloszynowska-Fraser, Laura Ansel-Bollepalli, Katy L. H. Cole, Angelica Foggetti, Barry Crouch, Gernot Riedel, Peer Wulff

**Affiliations:** 1 Institute of Medical Sciences, University of Aberdeen, Foresterhill, Aberdeen, AB25 2ZD, United Kingdom; 2 Department of Biochemistry and Molecular Biophysics, Columbia University, New York, NY 10032, USA; 3 Institute of Physiology, Christian Albrechts University Kiel, 24098 Kiel, Germany

## Abstract

Dysfunction of parvalbumin (PV)-positive GABAergic interneurons (PVIs) within the prefrontal cortex (PFC) has been implicated in schizophrenia pathology. It is however unclear, how impaired signaling of these neurons may contribute to PFC dysfunction. To identify how PVIs contribute to PFC-dependent behaviors we inactivated PVIs in the PFC in mice using region- and cell-type-selective expression of tetanus toxin light chain (TeLC) and compared the functional consequences of this manipulation with non-cell-type-selective perturbations of the same circuitry. By sampling for behavioral alterations that map onto distinct symptom categories in schizophrenia, we show that dysfunction of PVI signaling in the PFC specifically produces deficits in the cognitive domain, but does not give rise to PFC-dependent correlates of negative or positive symptoms. Our results suggest that distinct aspects of the complex symptomatology of PFC dysfunction in schizophrenia can be attributed to specific prefrontal circuit elements.

Functional imaging studies have suggested that dysfunction of the prefrontal cortex (PFC) underlies a multitude of deficits associated with schizophrenia, comprising positive (e.g. delusions and hallucinations), negative (e.g. emotional and social dysfunction) and cognitive (e.g. impairments in working memory and cognitive flexibility) symptoms^1^,^2^,^3^,^4^,^5^. Although these disturbances all appear to involve PFC dysfunction, it is unclear whether specific circuit elements within the PFC contribute differentially to symptom complexity.

Post-mortem studies of schizophrenia patients have suggested that PFC dysfunction involves alterations of the inhibitory circuitry of the PFC^6^,^7^,^8^. In particular reduced mRNA levels of the 67 kD isoform of the GABA synthesizing enzyme glutamic acid decarboxylase (GAD67) have been consistently reported^9^,^10^,^11^,^12^,^13^. These changes seem to dominantly affect a subclass of GABAergic interneurons, that expresses the calcium-binding protein PV, which itself shows reduced expression in schizophrenia^11^,^13^,^14^,^15^,^16^. In addition, genes associated with increased susceptibility to schizophrenia like DISC1, NRG1/ERBB4 or dysbindin, play crucial roles for circuit integration and function particularly of PVIs in rodents^17^,^18^,^19^,^20^,^21^. PVIs are also preferentially damaged by drugs of abuse such as phencyclidine and ketamine, which produce schizophrenia-like symptoms^22^,^23^. Accordingly impaired signaling of PVIs may form a common endpoint for different genetic and environmental factors leading to PFC malfunction and schizophrenia-associated deficits^19^,^24^.

In the isocortex PVIs mainly comprise basket and chandelier cells, which control network activity by targeting the soma and proximal dendrites or the axon initial segment of principal cells, respectively^25^. They are therefore well placed to exert rigorous control over PFC activity. However, little is known about the behavioral functions of these interneurons within the PFC circuitry.

To directly test how chronic impairment of PVI signaling affects PFC-dependent behavior, we selectively blocked output from PVIs in the mouse PFC by cell-type- and region-selective expression of tetanus toxin light chain (TeLC). To distinguish PFC-dependent behaviors which require intact PVI signaling from those that do not, we compared PVI-selective interference with non-cell-type-selective perturbations of the same circuitry in behavioral assays that were chosen to detect alterations that map onto symptom categories (positive, negative, cognitive) in schizophrenia.

## Results

### PV-cell-specific disruption of synaptic transmission in the PFC

**Figure 1:**
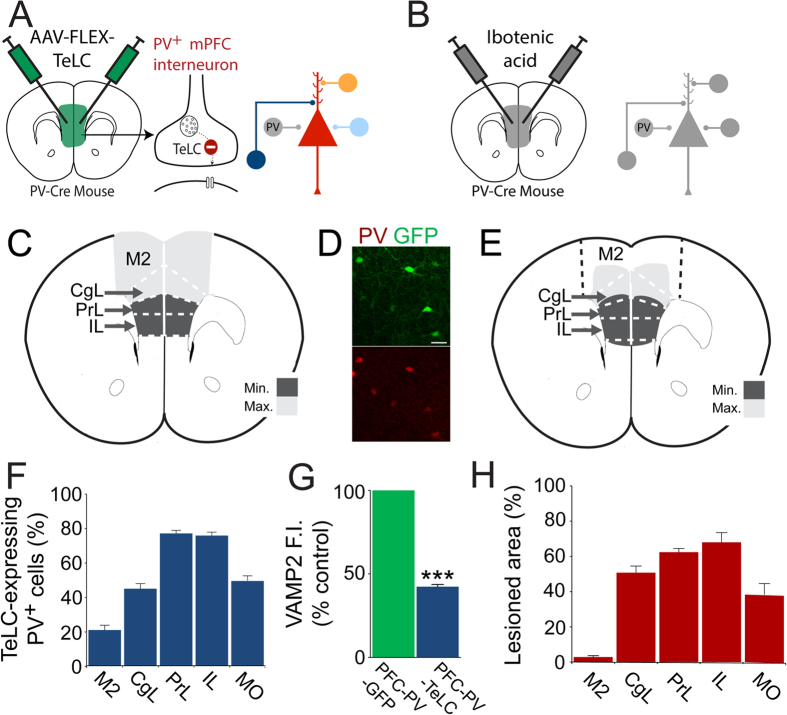
Cell-type-selective and general inactivation in the prefrontal cortex. (**A**) Injection of AAV-FLEX-TeLC into the PFC selectively blocks synaptic transmission in PVIs (grey shade in circuit cartoon) by cleaving VAMP2. Transmission in all other neurons remains intact. (**B**) Injection of ibotenic acid into the PFC causes general disruption of neuronal circuitry (grey shade). (**C**) Schematic of AAV spread in the mPFC of AAV-FLEX-TeLC-injected PV-Cre animals showing minimum (all animals >50% infected PVIs) and maximum (>50% infected PVIs only in most affected animals) infection area. (**D**) Immunohistochemistry illustrating co-localization of TeLC-GFP and PV in the mPFC (**E**), Schematic showing minimum (common to all animals) and maximum (in most affected animals) extent of ibotenic acid lesion in the mPFC. (**F**) Percentage of TeLC-GFP-expressing PVIs in subregions of the PFC. (**G**) Quantification of VAMP2 immunofluorescence intensity shows a substantial reduction in GFP-TeLC^+^/vGAT^+^ puncta in PFC-PV-TeLC animals compared with GFP^+^/vGAT^+^ puncta in PFC-PV-GFP animals. (**H**) Percentage of lesioned area in subfields of the PFC after ibotenic acid injection. PrL Prelimbic cortex; IL Infralimbic cortex; M2 Secondary motor cortex; CgL Cingulate; MO Medial orbital cortex. Scale bar in D = 20 µm. Data are mean ± s.e.m. ***p < 0.001.

We made stereotaxic injections of adeno-associated viruses carrying a GFP-tagged TeLC (or GFP alone as control, Fig. S1) reading frame inverted (3’ to 5’) in a flip-excision cassette (AAV-FLEX-TeLC and AAV-FLEX-GFP)^26^ into the PFC of PV-Cre knock-in mice^27^. As Cre-recombinase is required to flip the reading frame into the correct orientation, transcription of TeLC can only occur in PVIs^26^. Once expressed, TeLC efficiently stops transmitter release by cleaving VAMP2, a protein required for synaptic vesicle docking^26^ (Fig. 1A). Bilateral infusions of AAV-FLEX-TeLC resulted in expression of TeLC in over 75% of PVIs in prelimbic and infralimbic regions of the PFC (Fig. 1C,D,F). Additionally, TeLC expression was found in the cingulate and medial orbital cortices, demonstrating functional removal of PVIs across an area of mouse PFC thought to integrate functions of the dorsolateral, medial and orbital PFC in humans^28^,^29^,^30^. Transgene-expression in PV-negative cells was scarce (4.3%) and likely includes PVIs containing PV levels below detection threshold. Similar results were obtained for control AAV-FLEX-GFP injections (PFC-PV-GFP mice, data not shown). As expected, immunoreactivity for VAMP2 was strongly reduced in TeLC-positive terminals of PFC-PV-TeLC mice when compared with GFP-positive terminals of PFC-PV-GFP mice (*p* < 0.0001; *t* = 22.2; n = 4 animals per group; Fig. 1G). The residual VAMP2 immunoreactivity is likely due to low affinity recognition of VAMP2 cleavage products by the antibody^31^. However, using the same AAV-FLEX-TeLC we have previously shown that transmission of hippocampal PVIs was fully abolished 10 days after virus injection despite residual VAMP2 immunoreactivity^26^. In principle, loss of PVI-mediated inhibition could induce hyper-activity in pyramidal cells and induce ictal discharges in the PFC, which in turn could complicate the interpretation of behavioral data. However, local field potential (LFP) recordings from PFC showed no indication of enhanced synchronous activity in PFC-PV-TeLC mice. PFC-PV-TeLC and PFC-PV-GFP mice displayed similar absolute power at different spectral frequency bands (Fig. S2). In a second set of experiments we also made non-cell-type-specific PFC lesions, which have previously been used to investigate the behavioral role of the PFC, by injection of the fiber-sparing neurotoxin ibotenic acid^32^,^33^,^34^,^35^,^36^,^37^ (or saline as control; PFC-Lesion and PFC-Saline mice; Fig. 1B,E,H). We reasoned that this approach would confirm the PFC-dependence of our behavioral tests and that qualitative comparison of the behavioral deficits between PV-cell-selective and non-cell-type-selective circuit lesions would allow us to dissociate general PFC functions from those that specifically involve PVIs. To produce perturbations of comparable location and extent we titrated ibotenic acid injections according to viral spread.

### PVIs in the PFC are required for spatial working memory

**Figure 2:**
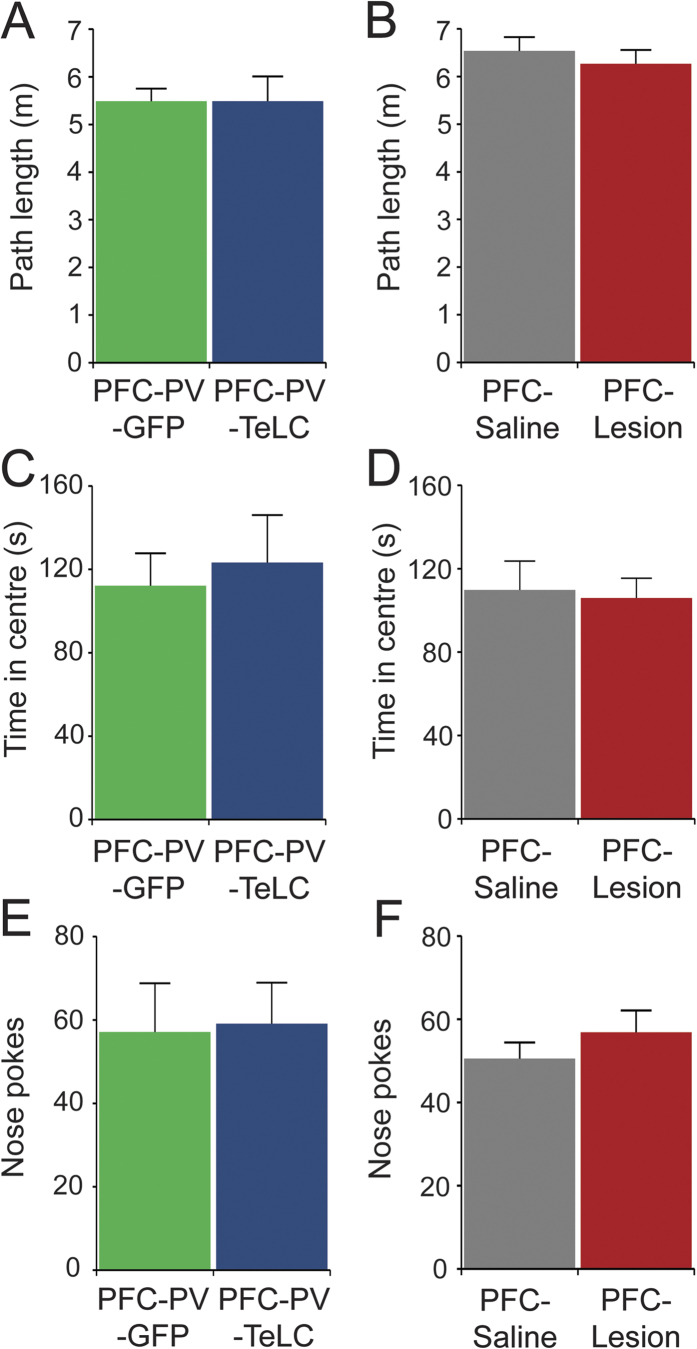
Neither PFC-PV-TeLC nor PFC-Lesion animals showed alterations during baseline behavioral assessment. Analysis of path length in the open field for (**A**), PFC-PV-GFP (n = 8) and PFC-PV-TeLC (n = 10) and (**B**), PFC-Saline (n = 9) and PFC-Lesion (n = 13) animals suggests normal baseline motor activity. (**C,D**) Time spent in the centre of an open field arena indicates no alterations in anxiety-related thigmotaxis. (**E,F**) The number of nose pokes during exploration of a hole-board arena is similar in all groups of mice indicating normal exploratory activity. Data are mean ± s.e.m.

**Figure 3:**
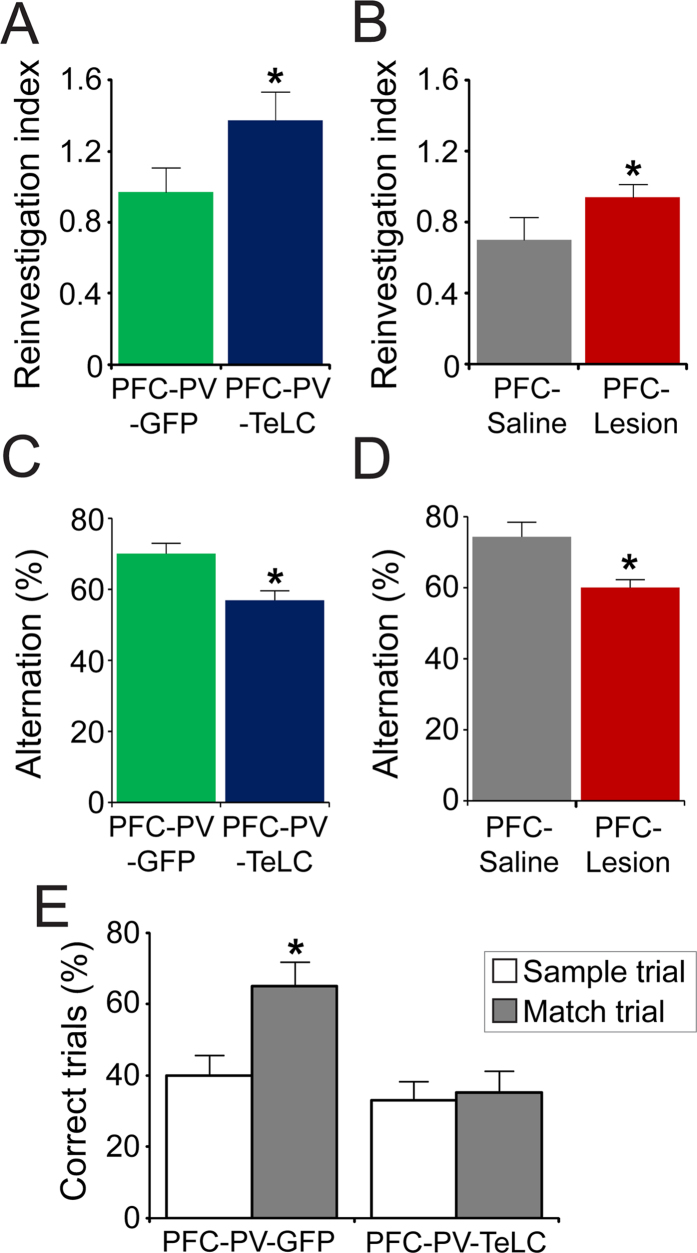
Working memory is impaired in PFC-Lesion and PFC-PV-TeLC animals. (**A**) PFC-PV-TeLC (n = 10) animals make significantly more repeat investigations in the hole-board test than PFC-PV-GFP (n = 8) and (**B**), PFC-Lesion (n = 13) mice make significantly more repeat investigations than PFC-Saline (n = 9) animals. (**C**) In the same cohorts of mice spontaneous alternation in a Y-maze is reduced in PFC-PV-TeLC animals and in (**D**), PFC-Lesion animals compared to respective controls. (**E**) The same PFC-PV-TeLC and PFC-PV-GFP mice were subjected to a match to place protocol in the water Y-maze. PFC-PV-GFP animals are able to retain the platform position during the 1 minute delay period, whereas PFC-PV-TeLC animals are not. Data are mean ± s.e.m. *P < 0.05.

Neither PFC-PV-TeLC nor PFC-Lesion mice showed obvious neurological deficits or alterations in locomotion and anxiety-related behavior (Fig. 2A-D). As deficits in working memory are a dominant cognitive symptom in both schizophrenia and PFC lesions^4^,^35^,^38^,^39^ we investigated the involvement of PVIs in the production of working memory. We implemented a hole-board-based working memory test by analyzing repeat investigations of previously explored holes. Although all groups of mice showed similar overall levels of nose pokes (Fig. 2E,F), indicating similar exploratory activity, both PFC-PV-TeLC and PFC-Lesion animals showed increased levels of repeat investigations (PFC-PV-TeLC vs. PFC-PV-GFP, *p* = 0.04; *t* = 1.97; PFC-Lesion vs. PFC-Saline, *p* = 0.04; *t* = 1.98) (Fig. 3A,B). This deficit was confirmed in a spontaneous alternation task in the Y-maze, where alternation levels were high in PFC-PV-GFP and PFC-Saline animals (70.0 ± 3.0% and 74.3 ± 4.1% respectively), but significantly lower in PFC-PV-TeLC and PFC-Lesion animals (56.9 ± 2.7% and 60.0 ± 2.3% respectively) (Fig. 3C,D). Both reinvestigation and spontaneous alternation probe a spontaneous on-line processing of information. To assess whether PVIs also contribute to temporally expanded forms of working memory, which are known to be impaired after PFC lesions^40^,^41^, we used a delayed (60s) match-to-place test in a Y-maze configuration in the water maze, where the percentage of correct responses in match trials represents trial unique short-term memory^26^. Whereas PFC-PV-GFP animals showed significant improvement in match trials, PFC-PV-TeLC animals did not (Fig. 3E).

Thus various modalities of PFC-dependent working memory critically depend on PVI integrity.

### Cognitive flexibility depends on prefrontal PVIs

**Figure 4:**
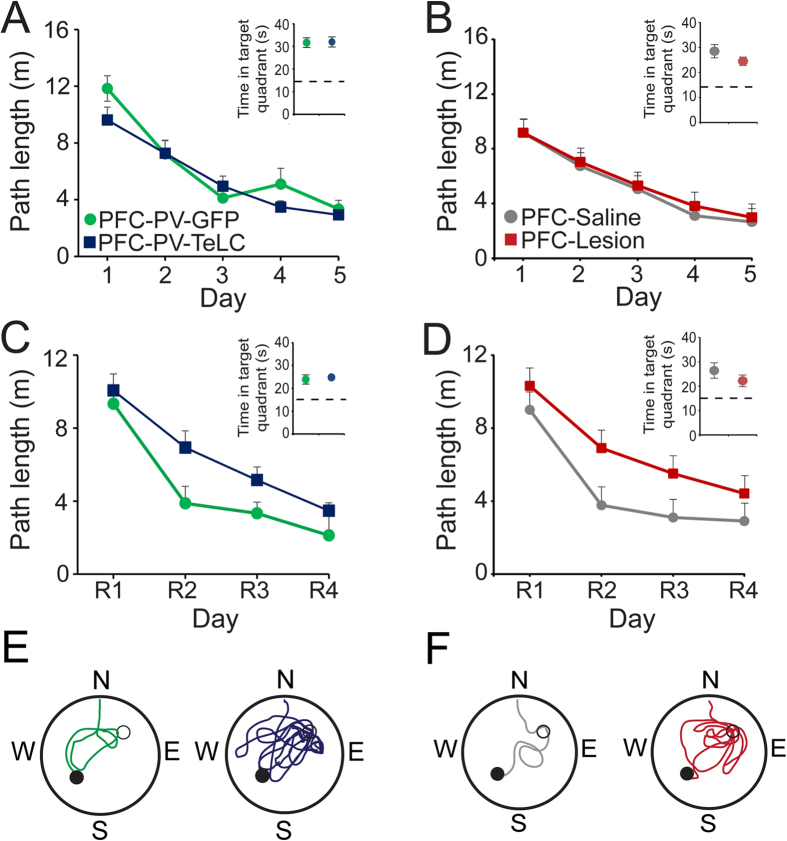
PFC-PV-TeLC and PFC-Lesion animals show impaired reversal learning. Both PFC-PV-GFP (n = 15) and PFC-PV-TeLC (n = 17) (**A**) and PFC-Saline (n = 9) and PFC-Lesion (n = 13) animals (**B**) learn the location of the hidden platform in an open field water maze as indicated by 1) the reduction in path length to platform over the 5 day training period and 2) the amount of time spent in the target quadrant in a probe trial after training (insets). (**C**) PFC-PV-TeLC and (**D**), PFC-Lesion animals show a deficit in reversal learning as indicated by a delay in path length reduction during 4 days of reversal training (R1-4). All groups spent significantly more time in the new target quadrant in a probe trial after reversal learning (insets in **C** and **D**). (**E,F**) Example traces of path taken by PFC-PV-GFP (**E**, green), PFC-PV-TeLC (E, blue), PFC-Saline (**F**, grey) and PFC-Lesion (**F**, red) animals on the third day of reversal training. The original platform location is indicated as a hollow circle, the new platform location as filled circle. Note the increased length around the original platform location of PFC-PV-TeLC and PFC-Lesion animals compared to respective controls. Data are mean ± s.e.m.

**Figure 5:**
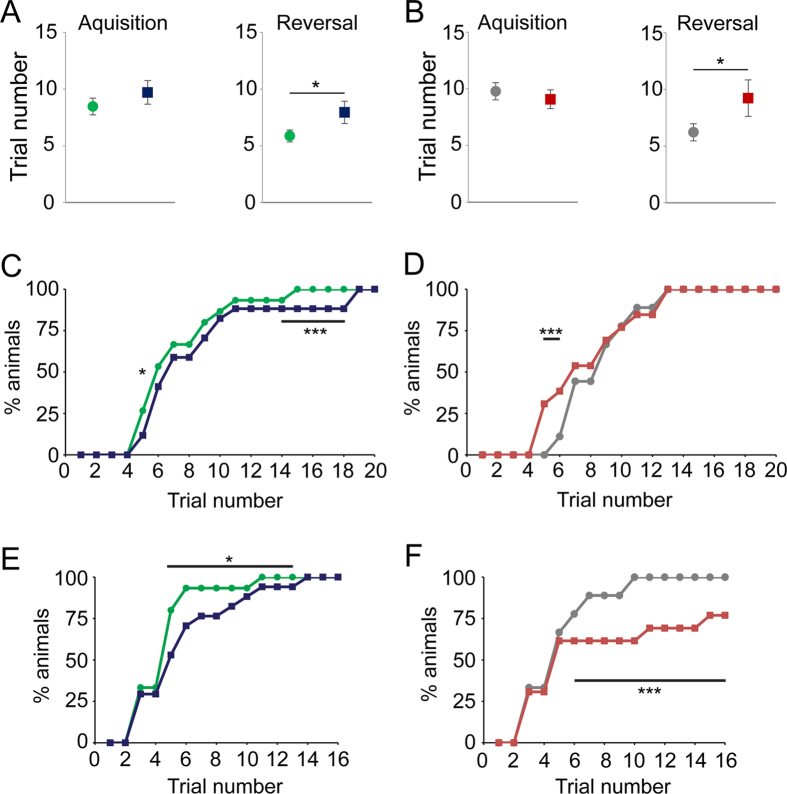
Criterion analysis confirms deficits in PFC-PV-TeLC and PFC-Lesion animals during reversal learning. The average number of trials to criterion for PFC-PV-TeLC (blue) (**A**) and for PFC-Lesion (red) animals (**B**) was similar to respective controls (green and grey) during acquisition (left) but significantly different during reversal (right) learning. (**C,D**) During initial water maze training PFC-PV-TeLC (**C**; n = 17) and PFC-Lesion (**D**); n = 13) animals show small differences when compared to their respective controls (n = 15 and 9), but all animals acquire the task to the same standard. (**E,F**) After re-location of the platform both PFC-PV-TeLC (**E**) and PFC-Lesion (**F**) animals are significantly impaired at learning the new platform location. Data are mean ± s.e.m. *P < 0.05; ***p < 0.001.

Cognitive flexibility is impaired in schizophrenia and after PFC lesion^41^,^42^,^43^,^44^. We investigated the relevance of prefrontal PVIs for cognitive flexibility in a reversal learning task in the water maze^36^,^37^. After initial spatial training (5 days, 4 trials/day) the platform was re-located to the opposite quadrant and mice underwent another 4 days (4 trials/day) of reversal training. During initial training PFC-PV-TeLC and PFC-Lesion mice learned the platform location as well as their respective controls as indicated by the reduction in path length (PFC-PV-TeLC/PFC-PV-GFP, day effect: F(4,120) = 45; p < 0.0001, no effect with group as factor: F’s < 1; PFC-Lesion/PFC-Saline, day effect: F(4,80) = 28; p < 0.0001; no effect with group as factor: F’s < 1) (Fig. 4A,B; see also Fig. 5) and the amount of time spent in the target quadrant during probe trials (*t’s* < 1.4) (Fig. 4 insets in A,B), indicating that spatial reference memory per se does not depend on the PFC^35^,^36^. In contrast, during reversal, PFC-PV-TeLC and PFC-Lesion mice needed more time to learn the new platform location although this was only significant for PFC-PV-TeLC mice (PFC-PV-TeLC vs. PFC-PV-GFP, day effect F(3,84) = 24; p < 0.001; group effect F(4,84) = 8.8; *p* = 0.006; and PFC-Lesion vs. PFC-Saline, F(3,60) = 2.1; *p* = 0.16) (Fig. 4C–F). The deficit was transient as PFC-PV-TeLC mice performed as well as controls in the probe trial after over-training (t’s < 1.1) (Fig. 4 insets in C,D). We confirmed these deficits by analyzing two additional parameters. 1) The number of trials to attain floor level (4m path length, derived from Fig. 4) in two consecutive trials was not different between PFC-PV-TeLC or PFC-Lesion groups and their controls (Fig. 5A,B; t’s < 1.4) during initial training, but was significantly higher in reversal trials (Fig. 5A,B, t’s > 1.7; *p*’s ≤ 0.05). 2) We established the percentage of subjects meeting criterion (4m path length) for each trial during both training phases (Fig. 5C–F). During initial training we detected small differences between PFC-PV-TeLC or PFC-Lesion animals and their respective controls (a mild delay or acceleration in learning, respectively; Fig. 5C,D). However, all subjects met criterion at completion of acquisition. During reversal both PFC-PV-TeLC and PFC-Lesion mice showed a deficit in learning the new platform location (Fig. 5E,F). However, whereas PFC-PV-TeLC mice met reversal criterion upon overtraining, more than 30% of PFC-Lesion mice failed to do so. These results suggest that PVIs contribute to prefrontal operations involved in the inhibition of previously learned responses.

### PVIs are dispensable for PFC-dependent behavior in a social preference test

**Figure 6:**
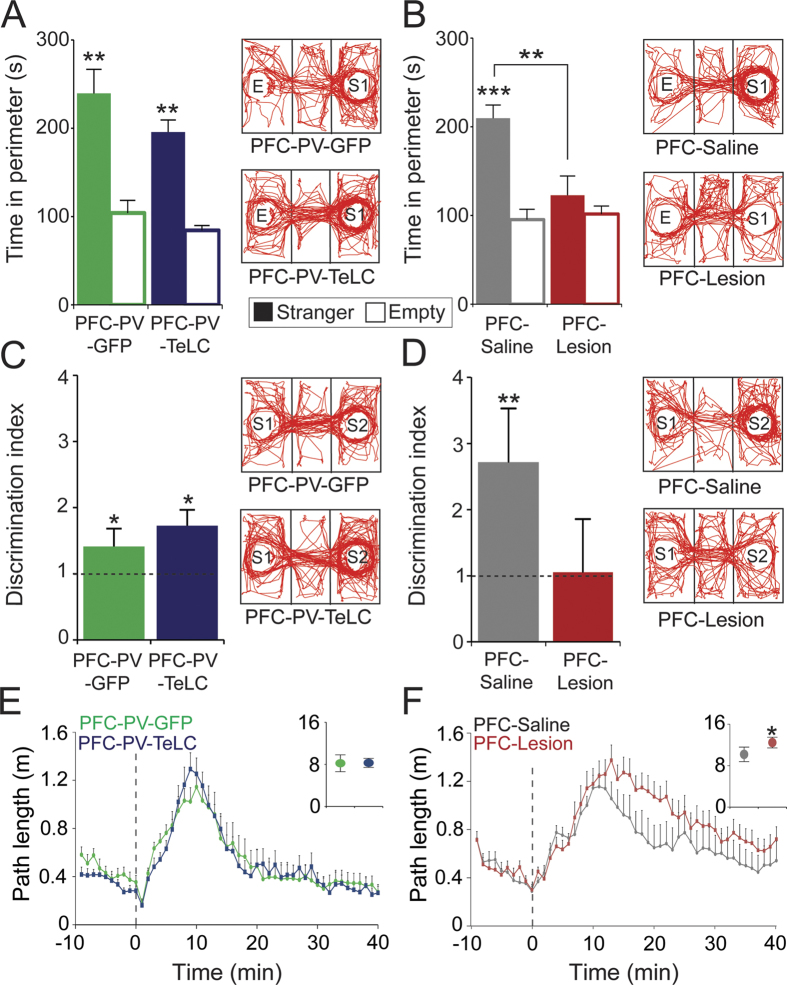
PFC-Lesion but not PFC-PV-TeLC mice show impaired social behavior and amphetamine hypersensitivity. (**A,B**) Quantification of time spent interacting with a conspecific stranger (S1) and an empty chamber (**E**) in a 3-chamber social preference test. (**A**) PFC-PV-GFP (n = 8) and PFC-PV-TeLC (n = 10) animals spent significantly more time interacting with a conspecific mouse than with an empty chamber. (**B**) PFC-Lesion animals (n = 13) do not show a significant preference for social interaction. Example traces in (**A**,**B**) illustrate the travelled path. Increased density in the stranger compartment of PFC-PV-GFP, PFC-PV-TeLC and PFC-Saline (n = 9) but not Lesion animals indicates preference for social interaction. (**C,D**) Discrimination between a novel (S2) and a familiar (S1) conspecific. PFC-PV-GFP, PFC-PV-TeLC and PFC-Saline animals spent significantly more time with the novel mouse indicating social recognition. PFC-Lesion animals did not distinguish between novel and familiar animals. Dashed line indicates level where equal time is spent with familiar and stranger animals. (**E,F**) Response to amphetamine. (**E**) Path length in an open field of PFC-PV-GFP (n = 8) and PFC-PV-TeLC (n = 10) animals. Dashed line indicates amphetamine administration. Both groups show enhanced locomotor activity after amphetamine administration. Insets show cumulated path length during the amphetamine activity period. (**F**) PFC-Saline (n = 9) and PFC-Lesion (n = 13) animals before and after amphetamine administration. Both groups show enhanced locomotor activity after receiving amphetamine but PFC-Lesion animals show a hypersensitivity as indicated by increased peak locomotor activity (inset) and slower decay kinetics. Data are mean ± s.e.m. *P < 0.05; **p < 0.01; ***p < 0.001.

Impairments in social function are common after prefrontal lesions and a component of the negative symptomatology in schizophrenia^2^,^45^,^46^. Similarly, interference with PFC function in rodents has been shown to reduce social interaction^47^,^48^. We tested PFC-PV-TeLC, PFC-Lesion and control animals for sociability and social recognition in a 3-chamber social preference test^49^. During sociability testing PFC-Lesion animals showed strongly reduced interaction with the conspecific animal (*p* = 0.003; t = 3.57) (Fig. 6B). However, PFC-PV-TeLC mice displayed similar levels of social interaction as PFC-PV-GFP control animals (*p* = 0.15; t = 1.59) (Fig. 6A). A similar pattern evolved in the second phase, where both control groups and PFC-PV-TeLC mice spent significantly more time with the unfamiliar than with the familiar animal (*p* = 0.003; *t* = 3.57 for PFC-PV-TeLC; *p* = 0.04; *t* = 1.97 for PFC-PV-GFP; *p* = 0.04; t = 2.00 for PFC-Saline), indicating intact social memory and a preference for social novelty (Fig. 6C,D). In contrast PFC-Lesion animals did not discriminate between familiar and unfamiliar mice (*p* = 0.3; t < 1) (Fig. 6D).

Accordingly social approach/avoidance behavior as well as social recognition memory/novelty preference depends on the PFC circuitry. PV-positive interneurons, however, do not seem to be a crucial component of this circuit.

### Impaired signaling of PV-positive PFC interneurons does not alter behavioral sensitivity to psychostimulants

Increased sensitivity to psychostimulants, such as amphetamine, is common in schizophrenia patients and considered a rodent correlate of the positive symptoms^45^,^50^,^51^. It is thought that both positive symptoms and increased amphetamine sensitivity are due to enhanced subcortical dopaminergic tone, which in turn correlates with and might be secondary to PFC dysfunction in schizophrenia^3^,^50^,^52^. Consistent with this idea, lesions of the PFC in rodents also increase amphetamine sensitivity and subcortical dopamine presumably through dysregulation of dopaminergic neurons in the ventral brainstem after loss of glutamatergic PFC afferent control^33^,^53^. Intraperitoneal injection of amphetamine caused a substantial increase in locomotor activity in all groups of mice with peak activity 10 to 15 minutes after injection (Fig. 6E,F). Compared with controls, PFC-Lesion animals showed significantly higher levels of locomotor activity (Fig. 6F), with increased peak activity (*p* = 0.04; t = 1.97) and slower decay kinetics (*p* = 0.005; t = 3.25), confirming the importance of the PFC for amphetamine sensitivity. In contrast, PFC-PV-TeLC animals did not differ from controls in amphetamine response (peak activity, t < 1; activity decay, t < 1) (Fig. 6E).

These results support the idea that the PFC controls subcortical dopamine release and may thus be causally involved in the generation of positive symptoms^53^. However, PVIs do not seem to be required for this regulatory function of the PFC.

## Discussion

Schizophrenia presents with a complex blend of positive, negative and cognitive symptoms and PFC dysfunction may be causally involved in the origin of all three symptom categories. Within the PFC, alterations of the inhibitory circuitry, which appear to preferentially involve PVIs, are among the most robust histological findings. To detect PFC functions that depend on intact signaling of PVIs we contrasted PV-cell-specific with general perturbations of the PFC circuitry. We found that PFC lesions produced behavioral alterations in all three symptom categories, whereas selective inactivation of PVIs caused deficits only in the cognitive domain.

Working memory represents a short-lasting on-line memory buffer that holds information relevant to ongoing tasks^54^. Although it is generally accepted that short-term memory relies on a distributed network of brain regions connected to and orchestrated by the PFC^35^,^55^, the physiological correlates of working memory are not clear. At the systems level synchronous network activity within the PFC and across functionally coupled brain structures may aid information processing during working memory^56^,^57^. Generation of network oscillations in turn is thought to depend on the activity of fast-spiking putative PVIs^58^,^59^,^60^,^61^. Here we show that PFC-dependent working memory indeed fully depends on intact transmission from PVIs.

Behavioral flexibility, which permits the individual to appropriate behavioral strategies to a changing environment, is another core function of the PFC that is impaired in schizophrenia^43^,^44^,^62^. The process may be mediated by PFC driven desynchronisation of network activity and thus functional uncoupling across task-relevant brain structures^63^. We have assessed behavioral flexibility in a water maze reversal task. Whereas both PFC-Lesion and PFC-PV-TeLC animals showed little difference in acquisition learning of a reference memory task, they needed more time to re-learn the platform position after its relocation. Analysis of reversal performance by subject revealed that this deficit was transient in PFC-PV-TeLC animals, where all mice eventually acquired the new location. By contrast, more than 30% of PFC-Lesion mice did not reach floor level after platform relocation suggesting that complete inactivation of the PFC circuitry induced more persistent impairments in reversal learning. Overall, our results indicate that impaired signaling of PFC PVIs leads to a transient failure to adjust behavioral responses to altered cue-reward associations.

Among the negative symptoms in schizophrenia social withdrawal and impairments in social cognition represent core elements and are amenable to modeling in rodents^2^,^46^,^64^. We found that general PFC dysfunction impairs both social-approach avoidance behavior and social recognition memory. Similar results were obtained after PFC perturbations in rats and in DISC1 and neuregulin-1 mouse models of schizophrenia (for review see^47^,^64^). Accordingly, optogenetic stimulation of the PFC has been shown to enhance social interaction in stressed mice^65^, presumably through prefrontal regulation of dopaminergic projections between ventral tegmental area (VTA) and nucleus accumbens and/or serotonergic raphe neurons^53^,^66^,^67^. Similarly, positive symptoms and enhanced amphetamine sensitivity in schizophrenia may result from deranged prefrontal control of brain stem dopamine neurons^3^,^33^,^50^,^52^,^53^. In line with this theory we found an enhanced peak and decelerated decay of the locomotor response to amphetamine in PFC-Lesion animals.

The results we obtained after non-cell-type-selective inactivation in mice thus confirm that PFC dysfunction may be causally involved not only in cognitive symptoms but also in the emergence of positive and negative symptoms, presumably by affecting the complex mono- and polysynaptic prefrontal regulation of subcortical monaminergic pathways^53^,^66^. Interestingly, complete disruption of GABA_A_-receptor-mediated inhibition in the PFC by bicuculline infusion has been shown to cause similar behavioral alterations as those we report here for PFC-lesion animals^68^,^69^,^70^. In contrast, selective inactivation of one sub-population of GABAergic interneurons – PVIs – caused deficits only in the cognitive domain, suggesting that these interneurons may subserve specific circuit functions in the PFC. Such a “division of labor” scenario between different populations of interneurons is further supported by our finding that analogous tetanus toxin-based targeting of a different, somatostatin (Sst)-expressing, population of interneurons in the PFC of Sst-Cre mice^71^ caused no change in working memory performance in the same task that was clearly impaired after inactivation of PVIs (Fig. S3).

In summary, impaired signaling of PVIs in the PFC, which is thought to form a core pathology in schizophrenia, may be causally involved in the development of cognitive symptoms. However, amphetamine-hypersensitivity and deficits in social-approach avoidance behavior as indicators of positive and negative symptoms, respectively, are not precipitated by PVI dysfunction. Based on these data we suggest that different aspects of the composite symptomatology of PFC dysfunction in schizophrenia can be allocated to specific neuronal elements.

## Materials and Methods

All procedures involving experimental mice were in accordance with the United Kingdom Animals (Scientific Procedures) Act 1986 and were approved by the Ethical Review Committee of the University of Aberdeen.

### Production of recombinant adeno-associated viral vectors

AAV vectors were produced as described previously^26^,^72^. Briefly, AAV virions were produced that contained a 1:1 ratio type 1 and type 2 capsid proteins by transfecting human embryonic kidney (HEK) 293 cells with the AAV backbone plasmids pAM-FLEX-TeLC or pAM-FLEX-GFP^26^ and AAV1 (pH21), AAV2 (pRV1) helper plasmids along with the adenovirus helper plasmid pFdelta6 using the calcium phosphate method^72^. 48 hours post transfection the cells were harvested and AAVs purified using 1 ml HiTrap heparin columns (Sigma) and concentrated using Amicon Ultra centrifugal filter devices (Millipore). Infectious AAV particles (viral titre) were calculated by serially infecting HEK293 cells stably expressing Cre-recombinase^26^ and counting GFP-positive cells.

### Animals, surgery and recording of local field potentials

Homozygous PV-Cre^27^ and Sst-Cre^71^ mice were purchased from Jackson laboratories (Repository number: PV 008069; SST 013044) and maintained as homozygous colonies. All stereotaxic injections and behavioral testing were carried out on male and female homozygous PV-Cre or SST-Cre littermates starting between P42 and P56. Animals were housed in a temperature-controlled environment with a 12 hour light/dark cycle and free access to food and water. Stereotaxic surgery was carried out as in^26^. Briefly, anesthesia was induced with 3% isoflurane in O_2_ by inhalation and maintained on 1.5–2% isoflurane throughout surgery. Heads of mice were fixed in a stereotaxic frame (Stoelting, USA), an incision was made on the skin above the skull and the skull exposed. Small holes were drilled relative to Bregma. The coordinates used were AP +2.0 mm, L ±1.0 mm, angle 17°, depth −2.6 mm. Lesions were made by injecting 1 μl of 1 mg/ml ibotenic acid, control animals received an injection of 1 μl vehicle (PBS). PFC-PV-GFP and PFC-PV-TeLC animals were injected with AAV-FLEX-GFP and AAV-FLEX-TeLC respectively. Both AAVs had a titer of about 6 × 10^6^ infectious particles per μl. 2 μl of rAAV along with 1 μl of 20% mannitol solution (total 3 μl volume) were injected over a 20 minute period. Stereotaxic injections were made using a pulled glass pipette (VWR) with an 8 μm bore width. After removal of the glass pipette the burr hole was filled with bone wax, the skin replaced and fixed using Vetbond tissue adhesive (3M).

For LFP recordings double twisted insulated gold wire depth electrodes (ADVENT Research Materials, Oxford, UK) were lowered bilaterally into prefrontal cortex (and an additional set into hippocampal CA1) at the same coordinates as viral deposition. Wires were externalized and a head-stage was formed of gold contact pins. Two gold plated watchmaker screws (TSE Systems GmbH, Germany) also connected to pins served as reference and ground respectively and were located above the posterior parietal cortex on either side of Lambda. Electrodes were anchored to the scull using dental cement and pins were covered with a low weight dummy connector.

Post-operative analgesia was given as needed. Behavioural analysis began 10 days after stereotaxic injections. All animals were housed individually after surgery and during behavioural testing periods.

Local field potentials (LFPs) from PFC were recorded while animals were freely moving in their holding cage. Low weight dummies were removed and replaced by a wireless data logger for continuous recording of 4 channels and on board memory (NeuroLogger, New Behavior, Zurich, Switzerland), set at a sampling rate of 200 Hz. A total of 10 minutes were recorded, files were extracted from the Neurologgers using the customised CommSW application and further converted into .txt format using an in-house MatLAB script (MatLAB 8.3, The MathWorks Inc., Natick, USA). Artefact free continuous recordings (120s) of LFPs were chosen and spectral power analysis conducted using Fast Fourier Transform in BrainVision Analyzer 2.0 software (BrainProducts GmbH, Germany) using ASCII (American standard code for information interchange) data format with multiplexed orientation, time domain, and sampling interval of 5000 μs (200 Hz).

### Histology

Mice were deeply anaesthetised by intraperitoneal injection of medetomidine/ketamine (1 and 6 mg per kg, respectively) and transcardially perfused with phosphate buffered saline (PBS) followed by 4% paraformaldehyde in PBS (PFA). After removal brains were post-fixed in PFA overnight at 4 °C. 50 μm coronal sections were taken on a VT1200S vibratome (Leica). Sections were permeabilised in 0.4% Triton X-100 for 30 minutes at room temperature and incubated with primary antibody overnight at 4°C in PBS containing 2% normal goat serum and 0.1% Triton X-100. Sections were washed three times in PBS containing 1% NGS for 10 minutes at room temperature and incubated with secondary antibody for 2.5 hours at room temperature. Sections were washed twice in PBS containing 1% NGS and once in PBS only for 10 minutes at room temperature, mounted on Superfrost glass slides (VWR International) and cover-slipped in Mowiol. Primary antibodies used were rabbit polyclonal GFP (1:1000) (Invitrogen), rabbit polyclonal PV (1:1000) (Swant), rabbit polyclonal VAMP2 (1:500) (Synaptic Systems), guinea pig polyclonal vesicular GABA transporter (vGAT; 1:500) (Synaptic Systems), mouse monoclonal GFP (1:1000) (Invitrogen). Secondary antibodies used were Alexa Fluor goat anti mouse 488, Alexa Fluor goat anti rabbit 488, Alexa Fluor goat anti mouse 647 (all Invitrogen; 1:1000) and goat anti rabbit Cy3 and goat anti guinea pig Cy5 (Jackson Immunoresearch; 1:500).

For analyses of GFP-TeLC-expessing PV-positive neurons (n = 9344 cells) sections were taken from at least 3 points along the rostro-caudal extent of the PFC and immunostained for GFP and PV. XY scans of the entire coronal section were taken on an epifluorescent Zeiss Axio Imager M1 microscope. Images of GFP and PV immunostaining were acquired separately and merged digitally in Adobe Photoshop (version CS3). The appropriate image of the mouse brain atlas^73^ was digitally overlayed to delineate the brain section into regions. The total numbers of PV-positive and GFP-TeLC-positive neurons were counted in the following regions: prelimbic cortex, infralimbic cortex, cingulate cortex area 1, secondary motor cortex, ventral orbital cortex, medial orbital cortex. Water maze experiments were replicates of two cohorts. Only cohort 1 was quantitatively assessed (n = 10/17). For cohort 2 and all PFC-PV-GFP animals correct viral expression was confirmed visually.

For analysis of ibotenic acid lesions, brains were flash frozen in liquid nitrogen and 40 μm sections were cut on a cryostat (Leica) and mounted on superfrost plus slides. Sections were stained with cresyl violet and images captured with a Zeiss Axioimager M1 microscope. The mouse brain atlas was digitally overlayed and the lesion in each brain area calculated as a percentage of the total brain area in ImageJ.

For analysis of VAMP2 expression three sections from four animals from both the PV-PFC-GFP and PV-PFC-TeLC groups were randomly selected and immunostained for VAMP2, vGAT and GFP. Images were taken using a Zeiss LSM 510 confocal microscope at equal exposure times for each image. Analysis was carried out blind to the experimental group. GFP or GFP-TeLC and vGAT-positive bouton-like structures were randomly chosen and traced using Zeiss Zen 2009 image software. Fluorescence intensity for VAMP2 within the traced area was measured as a mean grey scale value in ImageJ. In total 87 GFP-positive and 65 TeLC-positive puncta were analysed.

### Behavioural analysis

Behavioral testing of Experiment 1 was performed in PFC-PV-TeLC mice (n = 10) first to detail the phenotypes relative to PFC-PV-GFP (n = 8) mice. In a second follow-on experiment PFC-Lesion mice (n = 13) were tested against PFC-Saline mice (n = 9). This experimental design precludes a direct 2 × 2 statistical comparison. The general sequence of testing was open field, hole board, Y-maze alternation, social interaction, water maze and amphetamine challenge. Water maze experiments of PFC-PV-TeLC and PFC-PV-GFP mice were replicates of two cohorts with n = 7 for each group in the second cohort.

A separate cohort of PFC-Sst-GFP (n = 8) and PFC-Sst-TeLC (n = 11) was tested in a spontaneous alternation paradigm.

### Open field

Motor activity was analysed in a square white Perspex arena (60 × 60 cm). Animals were placed in the arena and tracked for 10 minutes with an overhead CCTV camera, data was recorded onto a PC and analysed using Ethovision XT software (Noldus). The arena was cleaned thoroughly between animals to eliminate odour cues. We analysed path length travelled during the trial, time spent in periphery or centre (equi-area) and habituation, as measured by change in path length over time.

### Hole-board and reinvestigation

The 40 cm by 40 cm hole board-arena was devoid of side walls and elevated 10 cm above the bench. It had 16 equi-distant holes containing infra-red sensors to record “nose-pokes” into the hole. The number of nose-pokes over a 5 minute period was recorded for each animal. Each trial was simultaneously recorded using an overhead CCTV camera for post-hoc analysis of reinvestigation. Reinvestigation was analyzed manually by an experimenter blind to the experimental group. The position of each nose-poke was noted, and the number of repeat pokes into previously explored holes was divided by the number of initial pokes. An animal that did not reinvestigate any holes would receive a score of 0.

### Match to place Y-maze

The match to place Y-maze was carried out as detailed in^26^. Briefly, trials were carried out in a circular white Perspex pool 150 cm in diameter and 50 cm deep filled with water to a depth of 35 cm with water at 21 ± 1 °C. The arena was equipped with 8 radiating arms (arm length 55 cm, arm width 15 cm, central area 40 cm diameter). The protocol was a delayed matching to place test with 3 trials per day (max trial time 60s) and inter-trial intervals of 60 s. The mice were released into the start arm facing the wall. The first trial was a free swim/extinction trial with both the start arm and one possible goal arm open but without an accessible platform. In the second trial, a second goal arm containing the submerged platform was open (sample trial) and the animal released again from the start arm and allowed to find the hidden platform. If it did not find the platform within 60s it was guided to it by the experimenter. Trial 3 was a match trial, where the animal was again released from the start arm and was monitored for performance with the platform remaining in the same position. A perfect trial was defined as the animal leaving the start arm and immediately entering the goal arm. Entering the wrong arm or re-entering the start arm was counted as errors. Training was continued on consecutive days until control animals reached a criterion of >70% correct match trials. Locations of arms and platform were varied pseudo-randomly each day to prevent spatial reference memory bias.

### Y-maze spontaneous alternation

Spontaneous alternation was analysed in a white Perspex arena consisting of three equally sized arms in a “Y” configuration. The animal was placed at the end of one of the arms facing the wall and allowed to freely explore the arena for 10 minutes. Each trial was recorded using an overhead CCTV camera for post-hoc analysis. Trials were analysed manually by an experimenter blind to the experimental group and the sequence of arm entries noted. A complete alternation was defined as an animal entering each of the three arms in sequence without re-entering a previously explored arm. Percentage alternation was calculated as the number of total complete alternations, divided by the total number of arm entries minus two and expressed as a percentage.

### Water maze and reversal learning

Open field water maze and reversal learning was carried out in a white Perspex pool 150 cm in diameter and 50 cm deep filled with water to a depth of 35 cm with water at 21 ± 1 °C. The extra-maze environment contained numerous spatial cues. On days one to five animals were trained to locate a hidden platform with four trials per day and an inter-trial interval of 30 minutes. Animals were released from a position at one of the four cardinal compass points in a pseudorandom fashion with one release from each point per day. Maximum trial length was 90 seconds, if an animal failed to locate the platform within the trial period it was guided to it by the experimenter. Following training on day 5 the animals received a probe trial, where they were released opposite the “platform” location and allowed to swim for 60 seconds. On day 8 the animals received a “refresher” day where they were again trained to the original platform location. On day 9 the platform location was switched to the quadrant opposite to the original location and training continued for three further days. Following training on day 12 the animals again underwent a probe trial. Throughout all trials animals were tracked via an overhead CCTV camera connected to the PC running Any-Maze (Ugo Basile) software. Primary parameters analysed were path length, time in target quadrant, swim speed. As a secondary parameter we calculated the number of trials required to attain floor level performance (2 consecutive trials with ≤4 m path length in each trial). It provides a best performance scenario and contingency plots reveal the percentage of mice that met floor level for each trial.

### Social interaction

Social interaction was examined essentially as described in^49^. The testing apparatus consisted of a three-compartment white Perspex box. Each compartment was 20 cm long, 42 cm wide and 22 cm high. Dividing walls were made from clear Perspex with small circular openings, 8 cm in diameter allowing free access to each chamber. The two outer compartments contained metal wire cages where conspecific animals were held, trials were recorded via an overhead CCTV camera connected to a PC and analyzed using Ethovision XT (Noldus). Test animals were first allowed a habituation period of 10 minutes where they could freely explore the apparatus without any stranger animals present. The test animal was removed from the apparatus for five minutes. Next the animals were tested for sociability, when a stranger junior conspecific (S1) was introduced to one of the side compartment cages (randomly selected and counterbalanced). The test animal was introduced to the central compartment and allowed to freely explore for 10 minutes. Time spent with the nose point of the test mouse in the immediate vicinity of the cage containing the stranger animal was taken as social interaction. The test animal was then removed from the apparatus for 5 minutes and a second stranger (S2) was introduced to the opposing cage, with the design counterbalanced so that stranger 1 could remain in the same position or move to the opposite location. The test animal was again introduced to the central compartment and allowed to freely explore for 10 minutes. Time spent with stranger 2 was divided by time spent with stranger 1 to give a discrimination index, where 1 (dashed line in Fig. 6C,D) indicates equal time spent with each stranger. The apparatus was cleaned thoroughly between animals to eliminate odor cues.

### Amphetamine challenge

Amphetamine challenge took place in the open field arena. Animals were placed in the centre of the arena and allowed to freely explore for 10 minutes. They were then given an intraperitoneal injection of 5 mg/kg D-amphetamine sulphate salt (Sigma-Aldrich) dissolved in saline. Animals were returned to the arena and monitored for a further 40 minutes, the arena was cleaned thoroughly between trials to minimise odor cues. Movements were recorded by an overhead CCTV camera and movements tracked using Ethovision XT software (Noldus).

### Statistical analysis

Statistical analyses were performed using Minitab version 15 (Minitab, Inc.) or GraphPad Prism 5.0. Unless otherwise stated data were analysed with two-tailed paired or unpaired Student’s t-test as appropriate. We also used two-way ANOVA with treatment as between-subject and trial/day as within-subject factors. Contingency analysis applied Fisher’s exact or Chi-Square tests. The null hypothesis was rejected for alpha greater than 5%.

## Additional Information

**How to cite this article**: Murray, A. J. *et al*. Parvalbumin-positive interneurons of the prefrontal cortex support working memory and cognitive flexibility. *Sci. Rep*. **5**, 16778; doi: 10.1038/srep16778 (2015).

## Supplementary Material

Supplementary Information
