# Supplementary material for: Parvalbumin-positive interneurons of the prefrontal cortex support working memory and cognitive flexibility
Source: Sci Rep. 2015 Nov 26;5:16778. doi: 10.1038/srep16778 (PMC4660359; doi:10.1038/srep16778)
Supplement: Supplementary Information [file srep16778-s1.docx]

**Supplementary Information**

**Parvalbumin-positive interneurons of the prefrontal cortex support working**

**memory and cognitive flexibility**

Andrew J. Murray, Marta Woloszynowska-Fraser, Laura Ansel-Bollepalli, Katy L. H. Cole, Angelica Foggetti, Barry Crouch, Gernot Riedel, Peer Wulff.

**Index of Supplementary Information**

**Figure S1.** AAV-FLEX-GFP injection into hippocampus causes no obvious behavioral deficits in the radial arm water maze.

**Figure S2.** EEG spectral analysis from local field potential recordings in the PFC of PFC-PV-TeLC and control animals.

**Figure S3.** Spontaneous alternation in a Y-maze is not affected in PFC-SST-TeLC mice.

**
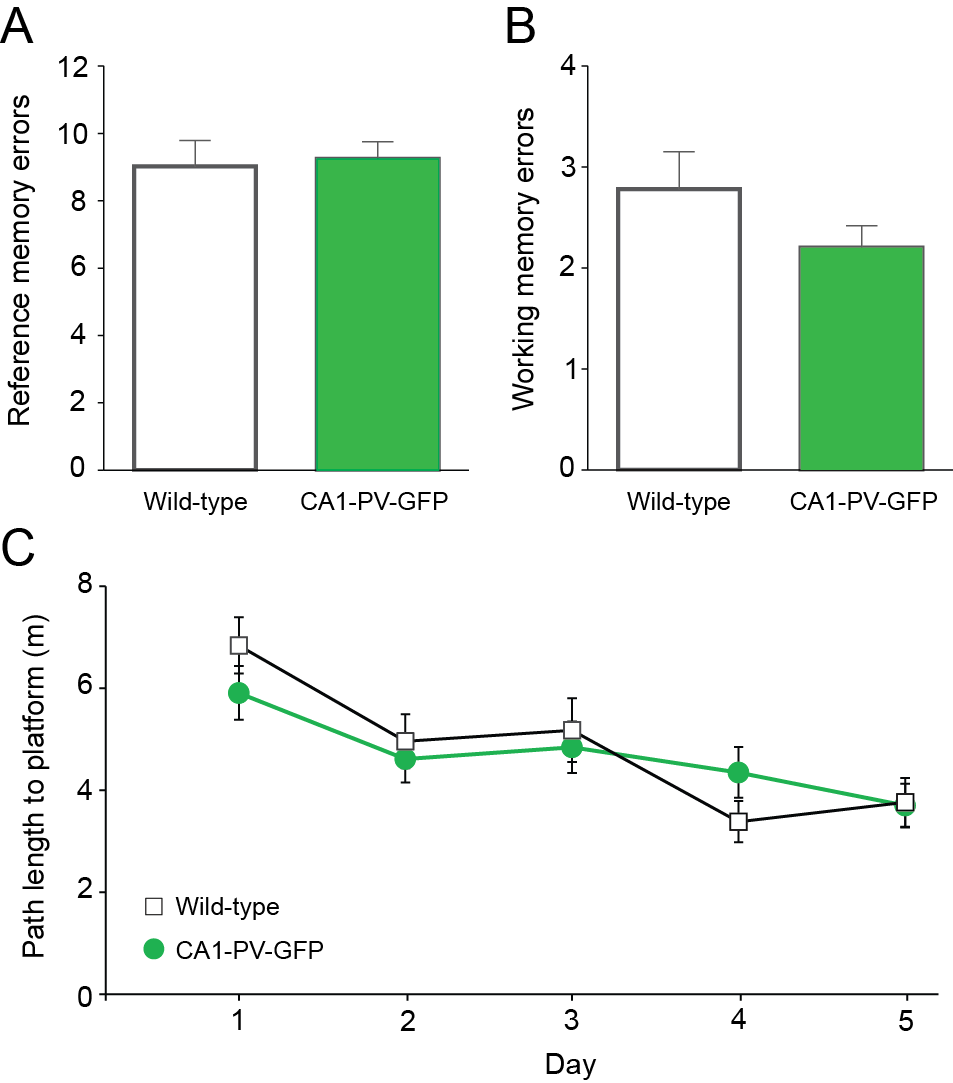
**

**Figure S1.** AAV-FLEX-GFP injection into hippocampus causes no obvious behavioral deficits in the radial arm water maze. PV-Cre mice with hippocampal injections of AAV-FLEX-GFP (n=7) did not differ significantly from un-injected wild-type mice (n=8) in ***A***, the number of reference or ***B***, the number of working memory errors made in the radial arm water maze. ***C***, The path length to platform in the radial arm water maze was also similar between groups. CA1-PV-GFP data are reproduced from Murray, A.J. et al. Parvalbumin-positive CA1 interneurons are required for spatial working but not for reference memory. Nat. Neurosci. 14, 297-299 (2011) with wild-type animals run concurrently to these experiments.

**
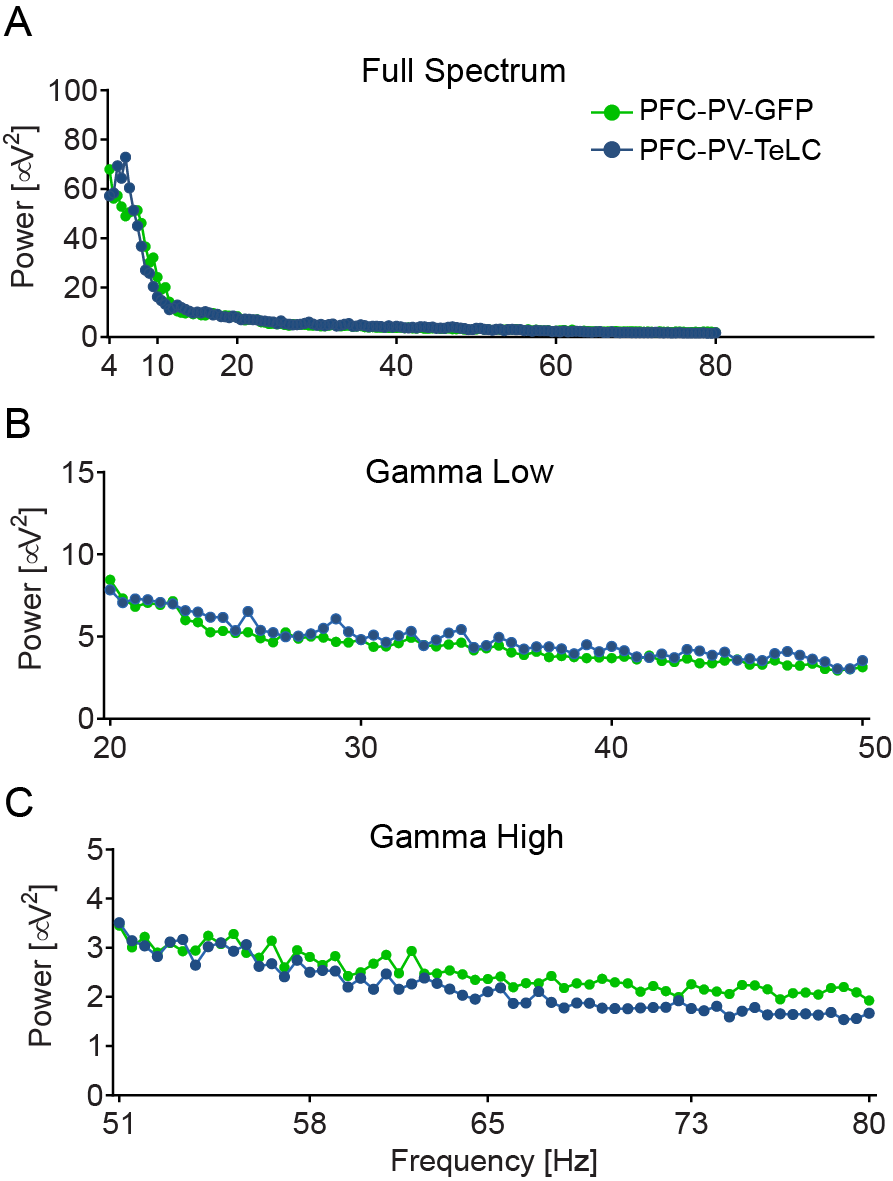
**

**Figure S2.** EEG spectral analysis from local field potential recordings in the PFC of PFC-PV-TeLC and control animals. Animals (PFC-PV-GFP n=7; PFC-PV-TeLC n=8) were equipped with bilateral depth wire-electrodes lowered into the limbic/prelimbic PFC for recording of local field potentials. Recordings were conducted in home cages and artefact free recordings of 120 sec were analysed in 1 sec bins using Fast Fourier Transform. The absolute power between cohorts did not differ over the complete spectrum (***A***) or low (***B***) and high (***C***) spectral gamma bands. All F’s < 1 for factor treatment. Data represent group means, SEM was omitted for clarity.


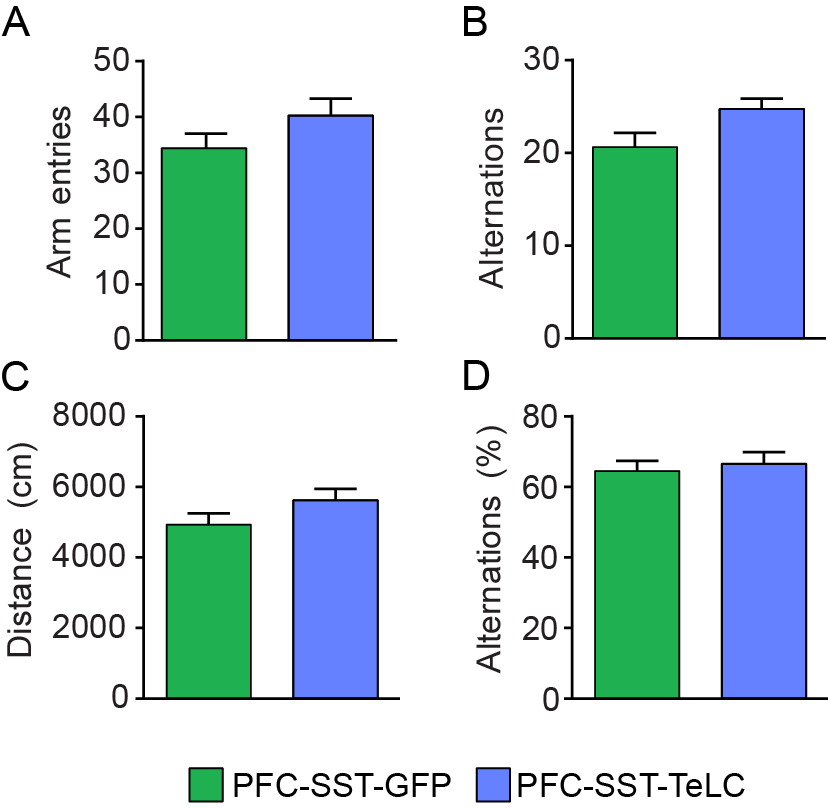


**Figure S3.** Spontaneous alternation in a Y-maze is not affected in PFC-Sst-TeLC mice. AAV-FLEX-TeLC or AAV-FLEX-GFP (as control) were injected into the PFC of mice expressing Cre recombinase under the control of the somatostatin (Sst) promoter to generate PFC-Sst-TeLC and PFC-Sst-GFP mice, respectively. After a recovery period of two weeks spontaneous alternation was investigated in the Y-maze. PFC-Sst-TeLC mice show a marginal and non-significant increase in activity revealed by a slightly higher number of arm entries (***A***), a higher number of alternations (***B***), and a longer distance covered during exploration (***C***). The critical measure for working memory, percent alternations (***D***), was also not different between both groups. Data represent mean ± s.e.m.
